# Latest Concepts in Endodontic and Periodontal Management of Diabetic Patients

**DOI:** 10.7759/cureus.52145

**Published:** 2024-01-11

**Authors:** Maryam Kuzekanani, Sara Mollamohamadi Kermani

**Affiliations:** 1 Endodontics, Endodontology Research Center, Kerman University of Medical Sciences and Health Services, Kerman, IRN; 2 Endodontics, Student Research Committee, Kerman University of Medical Sciences and Health Services, Kerman, IRN

**Keywords:** periodontal, management, endodontic, diseases, diabetic patients

## Abstract

The close interrelationship between chronic oral and dental inflammatory diseases, such as periodontal and apical abscesses (AA), with the patient's systemic condition, is one of the most interesting topics of study common between dentists and the medical staff. Chronic apical periodontitis and periodontal diseases are chronic infections of the oral cavity in which similar Gram-negative anaerobic microbiota are found and in both infectious diseases increased local inflammatory mediators^,^ levels may influence overall systemic conditions. One of the most important systemic diseases linked to periodontal, as well as AA, is diabetes mellitus (DM); likewise, the prognosis of chronic apical periodontitis and endodontic treatment is also associated with how DM is controlled in the patient. periapical and periodontal diseases may also contribute to the quality of DM control. DM affects many systems in the body, the most important; cardiovascular and renal systems, and this necessitates obeying several keynotes to provide safe endodontic and periodontal disease control in these patients that is the aim of this article to clarify for clinicians.

## Introduction and background

Historically, the provision of dental services focused heavily on the technical phases of dentistry, such as extractions, prosthetic replacements, caries management, and root canal therapy. Once the issue of focal infection came to the forefront in the early 1900s, a greater emphasis on the overall health of the patient came to bear on the dental profession and the potential for the interaction of oral disease, i.e., the role of microorganisms in the oral cavity and systemic disease was highlighted [1].

While heart disease and its impact on the patient was deemed a major concern [2,3], slowly and more recently there has been a focus on a wide range of diabetic conditions. Lifestyles that include obesity, inactivity, and the impact of aging have created a society that has faced the prevalence of type 2 diabetes, which enhances the risk for strokes, and cardiac as well as kidney diseases [3,4].

Due to the multiplicity of factors encountered in the diabetic state, along with the number of patients who suffer from diabetes, whether knowingly or unknowingly, the dental profession is challenged to not only manage patients suffering from this malady but also to educate the patient on the importance of regular oral health care [5]. Diabetes mellitus (DM) is a systemic disease consisting of several metabolic disorders resulting in chronic hyperglycemia due to impaired insulin secretion and/or insufficient tissue receptor response to serum insulin [6].

Diabetes is a serious challenge with serious human, social, and economic consequences worldwide, and according to American law is considered a disability the prevalence of which is constantly increasing [7]. According to the International Diabetes Federation (IDF), in 2013, the number of people with DM was 382 million, and in 2014 it was 387 million, which is 8.2% of the total adult population (20-79 years old) of the world. Moreover, DM has been the cause of more than 4.9 million deaths [8].

It is estimated that the number of affected people is currently around 422 million and is projected to rise to more than 592 million by 2035 [8,9]. Clinical reports show that 46% of people with diabetes are unaware of their disease, which can lead to more serious consequences [10].

Microangiopathy, nephropathy, neuropathy, macrovascular disease, and delayed wound healing are major disorders associated with diabetes. Moreover, periodontal tissue inflammation and involvement is the sixth most common diabetes-related disorder [11].

Periodontitis is an inflammatory and infectious disease caused by a group of anaerobic bacteria, including Porphyromonas gingivalis, Treponema denticola, Prevotella intermedia, Prevotella nigrescens, Eikenella corrodens, and Aggregatibacter actinomycetemcomitans, affecting teeth’s supporting tissues, such as the gums and alveolar bone, and leading to root caries secondary to gingival recession and root exposure [6,9,11-13].

Nowadays, a strong association has been found between the progression of periodontitis and several factors, including periodontal pathogens, high levels of proinflammatory cytokines, matrix metalloproteinases (MMPs), and low levels of inflammatory inhibitory cytokines [14,15]. The degree of tissue sensitivity and damage depends on the complex balance of cytokines produced by microorganisms. When the host's immune response is intensified, it can lead to tissue damage and the destruction of periodontal supporting tissues [14,16].

Systemic diseases such as diabetes can interfere with periodontal tissues and make the prognosis of the disease unfavorable [17]. Intravascular diffusion of microorganisms and their products into the body may occur following inflammation of periodontal tissues. In addition to periodontitis, other inflammations of the oral cavity, including tooth decay, salivary dysfunction, soft tissue pathology, dental infections, and sensory disturbances, have been associated with diabetes [18-20]. Before starting the treatment for a diabetic patient, the Endodontist should be aware of the important and specific considerations and the emergency treatments for these patients [21].

Successful management of diabetic patients begins with taking a complete history and review of systems. Clinicians should collect information about the patients’ recent blood glucose levels, at-home monitoring practices, and hypoglycemic and hyperglycemic episodes. Moreover, clinicians should be aware of the patient’s blood glucose control program, including intervals and dosages of received medications and special changes in their lifestyle [22].

Cortisol is an endogenous hormone that is released in the morning and under stress (e.g., during dental treatment), raising the patient's blood sugar; therefore, it is recommended that diabetic patients’ Endodontic treatment appointments be scheduled more often in the morning to reduce the risk of hypoglycemia [21].

For patients receiving injectable insulin, dental appointments should be scheduled so as not to interfere with maximal insulin activity because the risk of hypoglycemia is maximized during this period. If the treatment of these patients requires an invasive intervention or surgery, the dentist should consult the patient's physician regarding the adjustment of insulin dosage. At the beginning of each treatment session, the dentist should ensure that the patient is taking the appropriate medication and food and even measure blood sugar before beginning any treatment process to minimize the risk of a hypoglycemic episode. If the blood glucose is low, the patient should take an oral carbohydrate, and if it is high, treatment should be stopped, and the patient should be referred to a specialist to control blood glucose [21-23].

Moreover, considering appropriate local anesthesia for diabetic patients, especially those with neuropathy, is very important Local anesthetic solutions containing epinephrine are safe to use in all healthy and diabetic patients of both genders, except diabetic patients who have not taken their pre-operative hypoglycemic medication [21].

In addition, after completing dental treatment, the dentist should avoid prescribing aspirin or aspirin-containing compounds for these patients that may raise the risk of serious bleeding events. Since diabetic patients are prone to infections, any infection should immediately be treated and eliminated [21].

## Review

Diabetes and oral microorganisms

Unlike other cavities in the body, the oral cavity is not sterile. Due to direct and continuous contact with water, food, and air, it is a suitable environment for the accumulation and growth of microorganisms [24]. The surface of the oral cavity is covered with a wide range of microorganisms (bacteria, fungi, protozoa, and rarely viruses) that form biofilms and interact dynamically with the human body [25].

Because of this dynamic interaction, any disturbance in the balance between the human immune system and the type/density of the oral flora leads to disease in the soft and hard tissues of the oral cavity, including tooth decay and periodontal diseases [26-28].

Research shows that bacterial colonization and proliferation in the gingival tissue and the presence of gingival plaques are more common in diabetic patients compared with the control group. These bacteria include Aggregatibacter actinomycetemcomitans and Capnocytophaga ochracea [29], both opportunistic and anaerobic species that multiply in the gingival pockets as a result of severe hyperglycemia and destroy periodontal tissues by producing and releasing several enzymes [30].

Aggregatibacter actinomycetemcomitans is another bacterial species not commonly found in the oral cavity of healthy individuals and a study was seen in 43% of isolated samples from the oral cavity of diabetic patients [31]. This bacterium weakens periodontal tissue by destroying structural proteins, and if left untreated, it can lead to increased tooth mobility, damage to jawbone tissue, and tooth loss [32,33].

Some studies have shown that without timely treatment, the bacterium migrates to the heart through the bloodstream and colonizes the cardiac tissue [34,35]. Furthermore, recent studies have shown that patients with periodontal diseases are more likely to develop metabolic syndromes [36,37] and that the disease can exacerbate problems associated with hyperglycemia in diabetic patients [38]. This is because the inflammation caused by bacteria in the oral cavity and the involvement of periodontal tissue can lead to systemic diseases [39,40].

Diabetes and oral and dental disease

Poor glycemic control is associated with the development and progression of gingivitis, periodontitis, and alveolar bone destruction. The prevalence of periodontitis in diabetic patients has been estimated to be between 39% and 59.6% [41-43].

Moreover, periodontitis has been shown to be the leading cause of increased tooth mobility and loss in adults [44]. Changes in immune responses (for instance, neutrophil function disorders), gingival microflora, collagen structure and metabolism, vascular structure, gingival crevicular fluid, and genetic background are some of the factors involved in the two-way relationship between diabetes and periodontitis [45-47]

Controlling periodontitis can lead to an improvement in glycemic control by reducing inflammation and subsequently lowering insulin resistance. Antibody production in response to periodontal microorganisms and the presence of NK T lymphocytes, self-reactive B lymphocytes, positive acute-phase proteins, and autoantibodies induce an autoimmune state [48]. This autoimmune state in diabetic patients can destroy insulin-producing cells (β cells) and lead to hyperglycemia due to decreased insulin production [49]. The results of some studies suggest that the prevalence of dental plaque is higher in diabetic patients in comparison with those without diabetes [50]. Tooth decay is a chronic microbial disease caused by the destruction of the hard tissues of the teeth by lactic acid resulting from the fermentation of carbohydrates by lactobacilli (LB) and Streptococci mutans (SM) [51,52].

The prevalence of tooth decay varies based on race, sex, diet, oral hygiene, medications, and socioeconomic variables [53-57]; however, its overall range is estimated to be 84%-89% [28,56]. Diabetes is an independent risk factor for tooth decay. This is because chemical (glucose increase, pH decrease, calcium, phosphate, and fluorine decrease in saliva as a result of taking proline, and statins that are rich in protein. physical (change in flow) changes of saliva also lead to changes in the physicochemical properties of the oral cavity and make the environment conducive to candidiasis and colonization of pathogens that cause tooth decay [57-60].

Xerostomia is a common symptom in about 50% of diabetic patients suffering from reduced salivary flow [61]. This condition is more severe in patients with HbA1c levels higher than 8%. The role of diabetes in reducing salivary flow is still not well understood; however, it is suggested that angiopathy and neuropathy play an important role in salivary flow changes. Additionally, salivary flow can be influenced by other variables such as aging and drug prescriptions. Reduced saliva can reduce the production of antimicrobial mediators and increase the colonization of pathogens [61,62].

Burning sensation or dysesthesia in diabetic patients is associated with poor glycemic control, metabolic changes in the oral mucosa, angiopathy, candidiasis, and neuropathy [63]. Neuropathic pain in these patients may manifest as burning, pins and needles sensation, electric shock, or tingling, all of which are debilitating symptoms that have a significant effect on an individual’s physical and mental status that is associated with sleep disorders, anxiety, and depression [64].

An impaired sense of taste is likely in patients with poor glycemic control. A study found that 5.7% of diabetic patients had a sweet taste and 8.6% salty taste disorders [65,66]. Salivary disorders can be effective in changing the sense of taste or increasing the taste threshold; in addition, neuropathy is involved in increasing the taste threshold.

This sensory disturbance makes it difficult to maintain a balanced diet and weakens glycemic control [46,47,65]. Oral mucosal changes such as fissured tongue, geographic tongue, recurrent aphthous stomatitis, and some premalignant lesions such as oral lichen planus (OLP) are associated with diabetes [47,65,67].

The higher susceptibility of diabetic patients to oral changes is still debated; however, poor glycemic control, changes in the immune system, changes in blood flow in small vessels, reduced blood flow, xerostomia, and changes in the flow and composition of saliva have been reported to be effective in the occurrence of these symptoms.

Poor/delayed ulcer healing in soft and hard oral tissues is a well-known condition in diabetic patients with oral injuries [47,65]. According to some studies, delayed angiogenesis, impaired circulation, hypoxia, decreased innate immunity, decreased production of growth factors, and stress are among the factors affecting the delayed healing of oral ulcers in these patients [47,68].

Oral and dental treatment considerations for diabetic patients 

Hypoglycemia is the most common interfering problem in the oral and dental treatment of diabetic patients [22]. This risk is maximized during insulin activity pick when the patient does not eat before the dentist appointment, takes oral hypoglycemic medication, or when insulin secretion exceeds the body's need. Early signs of hypoglycemia include hunger, weakness, sweating, nausea, shakiness, irritability, and tachycardia [69].

Studies have shown that 15 g of glucose increases blood glucose by approximately 2.1 mmol/L within 20 minutes [70]. If a hypoglycemic episode is suspected, the dentist should stop dental treatment immediately and administer 15 g of oral carbohydrate via a candy, juice, or glucose tablet and monitor the patient’s blood glucose level to determine whether the administration of carbohydrates should be repeated. In case, the patient is unconscious or cannot swallow, the dentist should seek medical assistance and administer 20-50 mL of dextrose solution intravenously or 1 mg of glucagon via intravenous, intramuscular, or subcutaneous injection [71].

Since diabetes is a chronic condition, diabetic ketoacidosis (DKA) and hyperosmolar hyperglycemic state (HHS) are not considered emergencies in a dentist's office [22,72]. Because hyperglycemic patients show symptoms such as hunger, nausea, vomiting, weakness, or abdominal pain, it may be difficult for the dentist to distinguish between hyper and hypoglycemia [72].

Since taking small amounts of sugar does not cause a significant problem for a hyperglycemic patient, the dentist can presume such cases as hypoglycemic and immediately prescribe an oral carbohydrate source [22]. Real cases of hyperglycemia require medical intervention, and the patient should be given an insulin injection [21].

Dentists should consider the fact that diabetic patients, especially those whose blood sugar is not controlled, are prone to infection and delayed wound healing. Accordingly, appropriate antibiotic treatment should be provided depending on the type of dental treatment. In addition, if the dental treatment changes the patient's diet, the dentist should consult the relevant physician about adjusting the insulin dose and antibiotics. According to the AAE position statement in 2017 antibiotics are an important range of drugs in dentistry and their advantages include the resolution and prevention of the infection spread as well as minimizing the serious complications of infection. Also, according to this statement up to 50% of all antibiotics are used or prescribed incorrectly and this statement necessitates more consideration in the use of antibiotics in Endodontic and periodontal treatments [72]. It is also worth considering that salicylates can affect oral hypoglycemic compounds by increasing insulin secretion and sensitivity. Aspirin-containing medications should not be used for diabetic patients to prevent hypoglycemia [21,73].

## Conclusions

There is a close interrelationship between DM and endodontic and periodontal diseases. Periapical and periodontal infections impair the blood glucose level adjustment, on the other hand, DM intensifies periapical and periodontal diseases along with poor healing processes in these tissues. Further studies to evaluate the role of prescribing suitable antibiotics to better manage endodontic and periodontal diseases in diabetic patients are recommended considering antibiotic-resistance issues.
